# IQ as a moderator of outcome in severity of children’s mental health status after treatment in outpatient clinics

**DOI:** 10.1186/1753-2000-6-22

**Published:** 2012-06-07

**Authors:** Børge Mathiassen, Per Håkan Brøndbo, Knut Waterloo, Monica Martinussen, Mads Eriksen, Ketil Hanssen-Bauer, Siv Kvernmo

**Affiliations:** 1 Departments of Child and Adolescent Psychiatry, Division of Child and Adolescent Health, University Hospital of North-Norway, P.O. Box 19,, 9038, Tromsø, Norway; 2 Department of Psychology, Faculty of Health Sciences, University of Tromsø, 9037, Tromsø, Norway; 3 Division of Neurology, University Hospital of North-Norway, P.O. Box 19, , 9038, Tromsø, Norway; 4 RKBU-North, Faculty of Health Sciences, University of Tromsø, 9037, Tromsø, Norway; 5 Alta Child and Adolescent Mental Health Service, Finnmark Hospital Trust, P.O. Box 1294, 9505, Alta, Norway; 6 Department of Research and Development, Division of Mental Health Services, Akershus University Hospital, 1478, Lørenskog, Norway; 7 Centre for Child and Adolescent Mental Health, Eastern and Southern Norway, P.O. Box 4623, Nydalen, 0405, Oslo, Norway; 8 Department of Clinical Medicine, Faculty of Health Sciences, University of Tromsø, 9037, Tromsø, Norway

## Abstract

**Background:**

Psychotherapy is an effective treatment for mental health disorders, but even with the most efficacious treatment, many patients do not experience improvement. Moderator analysis can identify the conditions under which treatment is effective or whether there are factors that can attenuate the effects of treatment.

**Methods:**

In this study, linear mixed model analysis was used to examine whether the Full Scale IQ (FSIQ), Performance IQ (PIQ) and Verbal IQ (VIQ) on the Wechsler Intelligence Scale for Children – Third Edition, moderated outcomes in general functioning and symptom load. A total of 132 patients treated at three outpatient child and adolescent mental health services (CAMHS) were assessed at three different time points. The Children’s Global Assessment Scale (CGAS) and the Health of the Nation Outcome Scales for Children and Adolescents (HoNOSCA) were used to measure the severity of impairments in general functioning and symptom load. IQ was assessed at the start of treatment.

**Results:**

Moderator analysis revealed that the FSIQ × time interaction predicted changes in CGAS scores (*p* < .01), and that the PIQ × time interaction predicted changes in HoNOSCA scores (*p* < .05). The slopes and intercepts in HoNOSCA scores covaried negatively and significantly (p < .05). The same pattern was not detected for the CGAS scores (p = .08).

**Conclusions:**

FISQ and PIQ moderated change in general functioning and symptom load, respectively. This implies that patients with higher IQ scores had a steeper improvement slope than those with lower scores. The patients with the highest initial symptom loads showed the greatest improvement, this pattern was not found in the improvement of general functioning.

## Introduction

Numerous studies have shown that psychotherapy is an efficacious treatment for mental health disorders among children and adolescents [1], but even with the most efficacious interventions, many patients do not experience therapeutic improvement. In clinical decision-making, the ability to recognise these patients is crucial when determining which type of psychotherapy a patient should be offered. Moderator analysis, a method that can be used to examine this question, can reveal the conditions under which a treatment is effective or whether there are factors that are attenuating the effects of treatment [2]. Examples of factors moderating the effects of therapy are comorbidities, parental depression, a family’s need for public assistance and gender [3,4]. Developmental factors are recognised as a potentially important moderator of psychotherapy, but few studies have used measures of children’s and adolescents’ cognitive developmental levels to examine whether they have a moderating effect [5].

Low IQ is a risk factor for mental health disorders. Approximately one third of children with an intellectual disability have mental health disorders [6-9]. A study of Australian children also showed that borderline intellectual functioning (IQs in the range of 70–85) increased the risk of mental health disorders [10]. In spite of the finding that low IQ is a risk factor for mental health disorders, only a small number of studies have investigated whether patients’ IQs moderate the effects of therapy. In a study of cognitively based treatment of children with antisocial and aggressive behaviour, it was discovered that low IQs predicted a worse outcome for girls but not boys [11]. In the Multimodal Treatment Study of Children with Attention Deficit/Hyperactivity Disorder (ADHD), it was found that among children with severe ADHD whose parents had depressive symptoms, those with an IQ ≥ 100 responded better to both medical treatment and combined medical and behavioural treatment than those with an IQ < 100 [4].

The Health of the Nation Outcome Scales for Children and Adolescents (HoNOSCA) [12] and the Children’s Global Assessment Scale (CGAS) [13] are designed to assess different aspects of children’s mental health status. The HoNOSCA primarily measures symptom load, whereas the CGAS measures general functioning. Scores on the HoNOSCA and the CGAS are highly correlated [14,15], indicating that they measure much of the same construct. Several studies have found that both the HoNOSCA [14,16,17] and the CGAS [18] are able to detect changes in the mental health status of children with mental health problems. A positive correlation has been found between initial severity and changes in HoNOSCA scores, indicating that higher initial severity predicts larger changes in outcome [14,19].

Studies examining IQ as a predictor of HoNOSCA and CGAS scores have yielded mixed results. The results of a study of IQ as a predictor of HoNOSCA scores showed that IQ predicted an additional 6 % of the variance after controlling for age and gender [20]. Results from the same study revealed that IQ did not predict CGAS scores. A correlation has been observed between IQ and CGAS score among children admitted to a psychiatric inpatient unit [21]. Furthermore, low IQ predicted CGAS scores in the clinical range in a study comparing the offspring of depressed and non-depressed parents [22]. In a study of youth with schizophrenia and bipolar disorder at a psychiatric inpatient unit, no significant associations were found between IQ and HoNOSCA scores at admission or at follow up six years later [23]. To our knowledge, no study has reported whether a patient’s IQ moderates outcome in terms of general functioning and symptom load as measured by the CGAS and the HoNOSCA.

The objective of this study was to examine changes in symptom load and general functioning in an outpatient sample using the HoNOSCA and the CGAS as outcome measures. Participants were assessed with these instruments at three different time points. We also examined whether a patient’s cognitive functioning level (as assessed with the Wechsler Intelligence Scale for Children—Third Edition (WISC-III) IQ test) predicted changes in HoNOSCA and CGAS scores over these time points. We examined this relationship for both patients on a waiting list and patients in active treatment. Based on results from studies indicating that children with borderline intellectual functioning have a significantly increased risk for developing mental health problems compared to normally developing peers [10,24], we hypothesised that patients with higher IQ scores on the WISC-III IQ scales Full Scale IQ (FSIQ), Performance IQ (PIQ) and Verbal IQ (VIQ) would have better outcomes and faster improvements than patients with lower IQ scores.

## Methods

### Participants

The participants in this study (*N* = 132) were children and adolescents treated at three outpatient child and adolescent mental health services (CAMHS) in northern Norway. All eligible patients were consecutively asked to participate in the study. The mean age was 11.5 years (*SD* = 2.4*)*, 54.5 % (*n* = 72) of the sample was male and 45.5 % was female (*n* = 60). The clinical characteristics of the participants are presented in Table 1. Classified according to British norms [25], the proportion of Strength and Difficulties Questionnaire (SDQ) parent-rated measure of children with symptoms of mental health disorders in the abnormal/borderline range were as follows: emotional problems 47.9 %, conduct problems 37.1 %, hyperactivity 51.5 %, peer relationship problems 54.5 %, and problems with pro-social behavior 18.2 %.

**Table 1:** Descriptive Data for the Total Sample

**M**	***M (SD; n)***
HoNOSCA	
Intake	12.35 (5.29; *n* = 128)
Assessment	11.11 (4.41; *n* = 118)
Follow-up	7.91 (4.34; *n* = 97)
CGAS	
Intake	67.66 (11.17; *n* = 128)
Assessment	68.49 (10.22; *n* = 109)
Follow-up	75.28 (9.53; *n* = 94)
WISC-III IQ scores	
FSIQ	84.77 (19.02; *n* = 132)
VIQ	83.81 (17.45; *n* = 132)
PIQ	89.52 (20.92; *n* = 132)
SDQ parent-rated	
Emotional problems	3.38 (2.43; *n* = 132)
Conduct problems	2.33 (1.88; *n* = 132)
Hyperactivity	5.44 (2.86; *n* = 132)
Peer relationship problems	2.73 (2.04; *n* = 132)
Pro-social behaviour	7.58 (2.06; *n* = 132)
Total score	13.87 (5.84; *n* = 132)

### Measures

*The Wechsler Intelligence Scale for Children—Third Edition* (WISC-III), Norwegian version [26], is an intelligence test for children aged 6–16 years. The test consists of 13 subtests that are combined into three IQ scores: Full Scale IQ (FSIQ), Verbal IQ (VIQ), and Performance IQ (PIQ). Both the split-half and test-retest reliability of the WISC-III IQ scores are high (*r*_*xx*_ > .93) [27].

*The Strengths and Difficulties Questionnaire* (SDQ) [28] is a behavioural screening questionnaire designed for children and adolescents aged 3–16 years. It has been widely used for research in the Nordic countries [29]. There are separate SDQ forms for youths, parents and teachers. In this study, the parent version was used. Each form consists of 25 items divided into the following scales: emotional symptoms, conduct problems, hyperactivity/inattention, peer relationship problems and pro-social behaviour. The factor structure of the SDQ has been replicated using confirmatory factor analysis in a sample of Norwegian children [30].

*The Children’s Global Assessment Scale* (CGAS) [13] is a rating scale that measures general functioning in children aged 4–16 years with a range from 1 (needs constant supervision) to 100 (superior functioning). The most impaired level of functioning during the previous month was rated. The CGAS has been evaluated in several studies and is widely used to assess the severity of mental health problems and outcomes [18,31]. A study of inter-rater reliability [32] among clinicians working in the Norwegian outpatient CAMHS revealed an intraclass correlation coefficient (ICC) of .61. A similar ICC was found in a comparable cross-national study [33].

*The Health of the Nation Outcome Scales for Children and Adolescents* (HoNOSCA) [12] consists of 15 scales that are rated from 0 (no problem) to 4 (severe to very severe problem) by clinicians. In this study, only the first 13 scales were used, and the total score was used as a measure of the overall severity of mental health problems (range 0–52). The HoNOSCA has been evaluated in several studies and has been found to be easy to use, reliable, valid and sensitive to change [14,16,17,32-34] A study of inter-rater reliability [32] among clinicians working in the Norwegian child and adolescent mental health service revealed an ICC of .81, and in a comparable cross-national study [33], the ICC was found to be .84.

### Procedures

The participants were assessed by clinicians using the HoNOSCA and CGAS at three different time points: at the intake session (T0), at the start of treatment (T1) and after 6 months of treatment (T2) or at the end of treatment if treatment lasted less than 6 months. The mean waiting list time (the number of days from T0 to T1) was 140.5 days (*SD* = 70.1), and the mean treatment time (the number of days from T1 to T2) was 179.3 days (*SD* = 71.4). The WISC-III assessment was performed at T1.

There was some variation in the time at which the parent SDQ was completed: 77.3 % (*n* = 102) of the questionnaires were filled in at T1 and 33.7 % (*n =* 30) at T0. Examinations of differences between the questionnaires completed at T0 and T1 were performed with independent sample t-tests independently for scales measuring emotional symptoms, conduct problems, hyperactivity/inattention, peer relationship problems and pro-social behaviour. There was a small significant difference (*t*(130) = 2.59, *p* = .01, *r* = .22) between emotional scales completed at T0 (*M* = 4.37, *SD* = 2.79) and T1 (*M* = 3.09, *SD* = 2.24). No significant differences were found for the other scales.

This study was carried out in ordinary outpatient clinics with an unscreened patient population. The clinics had an eclectic approach to therapy and interventions, and were staffed with clinical psychologists, child psychiatrists and social workers. Most of the employees were specialist in their profession or under specialization. The specialization programs for psychologist and physicians last for five years after six years of university education, and includes compulsory seminars and supervision from a specialist.

This study was approved by The Regional Committee for Medical and Health Research Ethics, North Norway.

### Statistical analyses

All statistical analyses were performed using SPSS version 16.0. Some participants assessed at the intake session had missing data at later time points, and there were some differences in assessment time points. Repeated measures analyses of variance or regression analyses with dummy variables would have necessitated the exclusion of participants with missing data. Additionally, these statistical methods assume that all participants have been assessed at the same time points. To overcome these problems, linear mixed model analyses were used [35]. In a repeated measures design analysed with linear mixed model statistics, participants with missing data can be included in the analysis, the time points of assessment can vary and it is possible to specify the best variance-covariance structure for the data [35]. The results can be interpreted in the same way as a regression analysis results.

To test whether there were differences between the HoNOSCA and CGAS scores at different time points, time was treated as a fixed factor. Bonferroni post-hoc comparisons were used to adjust for multiple comparisons. The effect size of the different time points was examined by calculating *r* based on the results from an independent samples *t*-test. An interpretation of the effect sizes was performed according to the guidelines suggested by Cohen [36]. Effect sizes of *r* = .10 were interpreted as small, *r* = .30 as moderate, and *r* = .50 as large.

The models examining repeated HoNOSCA and CGAS measures, with the different WISC IQ scales as moderator variables, were constructed in a stepwise fashion. To test whether entering new variables into the model increased the model fit, changes in −2 log likelihood were used. The differences were examined with chi-squared statistics. The first independent variable entered in the model was time (the three time points) and the next variable was the WISC IQ score. Entering the different FSIQ-, PIQ- and VIQ-time interaction terms as the final variable in the mixed-model analysis was performed to examine the different WSIC IQ as moderators. The repeated measures of HoNOSCA and CGAS were entered at level 1 (data for individual patients) in the model, whereas the different WISC IQ scales were entered at level 2 (differences between patients). Time and the IQ scales were treated as covariates. An unstructured covariance structure was used.

## Results

### Characteristics of the sample

Of the 132 participants included in the study, 26.9 % (*n* = 35) dropped out or had missing data before the final HoNOSCA assessment was completed. We examined whether the patients with missing data were different from those with full data sets. To accomplish this, we compared patients with and without HoNOSCA scores at the final assessment with independent sample t-tests examining the following variables: HoNOSCA and CGAS score at the intake assessment, age, FSIQ, VIQ, PIQ and mental health as measured by the SDQ questionnaire. An examination of differences in gender distribution was performed with a Pearson chi-squared test. There were no significant differences between the participants with and without complete data from all assessments. This result indicates that even though some of the participants dropped out or had missing data after the first assessment, the results from the final assessment are representative of the whole sample.

### Symptom load measured by HoNOSCA

The mean HoNOSCA scores for the three different time points were significantly different (*F*(2, 340) = 24.60, *p* < .01). Time predicted changes in HoNOSCA scores (*b* = −2.16, *t*(112.70) = −8.40, *p* < .01). The different WISC IQ scores were examined in separate models. Neither entering the FSIQ×time (*χ*^*2*^ (1) = 2.04, *p* > .05) nor the VIQ×time (*χ*^*2*^ (1) = 0.09, *p* > .01) interactions did increased the model fit. Adding the PIQ×time interaction (*χ*^*2*^ (1) = 5.01, *p* < .05) did increase model fit, indicating that PIQ moderated the outcome in HoNOSCA scores. The interaction term PIQ × time predicted changes in the HoNOSCA score (*b* = −0.03, *t*(115.14) = −2.28, *p* = .02).The parameters of the final model are presented in Table 2.

**Table 2:** Parameters in the final models with HoNOSCA and CGAS as dependent variables

	***HoNOSCA as dependent variable***			***CGAS as dependent variable***		
	*b*	*SE b*	*95 % CI*	*b*	*SE b*	*95 % CI*
*FSIQ as moderator*						
Time	−2,16**	0,26	−2.66,-1.64	−2.15	2.68	−7.47,3.17
FSIQ	−0,07**	0,02	−0.10,-0.33	−0.07	0.07	−0.21,0.08
FSIQ × Time	-	-	-	0.07*	0.03	0.01,0.13
*PIQ as moderator*						
Time	0,30	1,11	−1.90,2.50	3,74**	0,59	2.57,4.91
PIQ	−0,01	0,03	−0.07,0.05	0,10**	0,03	0.03,0.17
PIQ × Time	−0,03*	0,01	−0.05,-0.00	-	-	-
*VIQ as moderator*						
Time	−2,15**	1,73	−2.67,-1.64	3,74**	0,59	2.57,4.91
VIQ	−0,05	0,02	−0.09,-0.01	-	-	-
VIQ × Time	-	-	-	-	-	-

Note: * p < .05, ** p < .01.

Post-hoc comparisons indicated that the mean HoNOSCA score at T0 (*M* = 12.35, *SD* = 5.29) was significantly (*p =* .01) higher than that at T1 (*M* = 11.11, *SD* = 4.42) and that the score at T2 (*M* = 7.91, *SD* = 4.42) was significantly (*p* < .01) lower than that at T1. The effect size of the reduction of HoNOSCA scores from T0 to T1 for the whole sample was small and non-significant (*r = .*12; *t*(237,78) = 1.95, *p* = .06), whereas the reduction from T1 to T2 was moderate (*r* = .34; *t*(209) = 5.17, *p* < .01).

The change in HoNOSCA scores across the three time points showed a significant variance in intercepts (*var(u*_*0j*_*)* = 29.12, *p* < .01) but not in slopes (*var(*_*u1j*_*)* = 2.30, *p* = .06) across participants. The slopes and intercepts covaried negatively and significantly (*cov(u*_*0j*_*, u*_*1j*_*)* = −7.14, *p* = .01). This result indicates that the participants with the highest initial HoNOSCA scores showed a greater improvement across the time points compared to the participants with the lowest scores.

### General functioning measured by CGAS

The mean CGAS scores for the three different time points were significantly different (*F*(2, 328) = 16.43, *p* < .01). Time predicted changes in CGAS scores (*b* = −3.74, *t*(104.11) = 6.33, *p* < .01). The different WISC IQ scores were examined in separate models. Neither entering the PIQ×time (*χ*^*2*^ (1) = 3.53, *p* > .05) or the VIQ×time (*χ*^*2*^ (1) = 2.66, *p* > .01) interactions did increased model fit. Adding the FSIQ×time interaction (*χ*^*2*^ (1) = 8.63, *p* < .01) did increase model fit, indicating that FSIQ moderated the outcome in CGAS scores. The interaction term FSIQ × time predicted changes in the CGAS score (*b* = 0.46, *t*(107.28) = 1.86, *p* < .01). The parameters of the final model are presented in Table 2.

Post-hoc comparisons indicated that the mean CGAS score at T0 (*M* = 67.66, *SD* = 11.17) was not significantly (*p =* 1.00) different from that at T1 (*M* = 68.49, *SD* = 19.22) and that the score at T2 (*M* = 75.28, *SD* = 9.53) was significantly (*p* < .01) higher than that at T1. The effect size of the increase in CGAS from T1 to T2 was moderate (*r* = .32; t(201) = −4.87, *p* < .01).

The change in CGAS scores across the three time points showed significant variance in intercepts (*var(u*_*0j*_*)* = 118.38, *p* < .01) across participants. There was no significant variance in slopes (*var(*_*u1j*_*)* = 9.02, *p* = .20) or in the covariation between slopes and intercepts (*cov(u*_*0j*_*, u*_*1j*_*)* = −27.25, *p* = .08).

## Discussion

The main objective of this study was to examine if the different WISC-III IQ scales moderates changes in symptom load and general functioning among children and adolescents referred to mental health outpatient clinics. The results indicated that the patients’ symptom loads and general functioning, as measured by HoNOSCA and CGAS, respectively, improved for the entire sample.

Symptom load showed a decrease from the start of treatment to the 6-month follow-up assessment, and the effect size of this change was moderate. The patients with the highest initial HoNOSCA scores showed the greatest improvement. This result is consistent with previous research [14]. The results indicated that PIQ moderated changes in HoNOSCA from the intake session to the follow-up assessment, indicating that the improvement slopes for patients with high PIQ were steeper than those with lower PIQ. There were no gender differences in the moderating effect of PIQ. FSIQ and VIQ did not moderate the outcome in HoNOSCA scores.

General functioning, as measured by the CGAS, improved from the start of treatment to the 6-month follow-up assessment. The effect size of this change was moderate. There was no significant variance across the participants in the intercept or slope of the change in CGAS scores across the measurements performed at the intake session, start of treatment or at the 6-month follow-up assessment. The results indicated that FSIQ moderated changes in CGAS scores and imply that the general functioning improvement slope for patients with high FSIQ were steeper than those with lower scores. There were no gender differences in the moderating effect of FSIQ. PIQ and VIQ did not moderate outcome.

In addition to psychometrical differences between the HoNOSCA and CGAS scales, distinct properties of the WISC-III IQ-scales may explain the differences in the predictability of outcome. In addition to measuring different cognitive abilities, the heritability of the WISC-III IQ-scales is dissimilar. The heritability of FSIQ, VIQ and PIQ in early adolescent is 65 %, 51 % and 72 %, respectively [37]. The environments influence the development of the WISC-III IQ score in different ways. Common environment influences on FSIQ, VIQ and PIQ in early adolescent are 18 %, 26 % and 0 %, respectively [37].

Although the CGAS and the HoNOSCA measure different aspects of mental health impairment, there is a large correlation between these measures [15]. This correlation indicates that the CGAS and the HoNOSCA measure much of the same psychological construct. In our study different WISC-III IQ scales moderated the outcome in general functioning measured and symptom load measured with the CGAS and the HoNOSCA. This difference could be explained by the different construction of the HoNOSCA and the CGAS. The HoNOSCA total score is the sum of 13 scales, including one question related to scholastic and language skills, which are areas that have a high correlation with IQ [38], whereas the CGAS consists of just one scale.

The identification of IQ as a moderator of changes in general functioning and symptom load does not explain the mechanism behind the relationship between IQ and outcome. The cognitive reserve model has been proposed as an explanation for the association between IQ and the development of mental health disorders [25,39]. This model postulates that “cognitive reserve” (CR), operationalized as, for example, education, occupational attainment and IQ, is a proxy measure of brain reserve capacity [40]. It could be that in addition to explaining the increased risk for mental health disorders, the CR model also explains why the patients in our study with higher IQ scores had a larger improvement in general functioning than the patients with lower scores. Because IQ is associated with both brain size [41] and other neuroanatomical and neurophysiological factors [38], it could be that the patients with the largest cognitive reserve had a greater capacity to benefit from the help they received at the outpatient clinic.

### Study limitations

The rate of dropout and missing data in this study was 26.5 %. Compared to other clinical studies, this was a small dropout rate. A meta-analysis across 125 studies of psychotherapy revealed a mean dropout rate of 46.9 % [42] Even if there were no significant differences between the participants with complete and missing data, there could potentially be relevant differences between these groups that were not examined.

The main methodological strength of our study is that it is carried out in ordinary outpatient clinics without low IQ as an exclusion criterion. In mental health research most outcome studies have been conducted under controlled experimental conditions with strict sample control selection [43]. This limits the external validity of the results. The methodological strength of this study is also the main limitation. In an ordinary outpatient clinic with an unselected patient population it is difficult to obtain information about the reason for dropout, an exact overview of the number of eligible patients, therapist competence and caseload, type of intervention and other potential relevant factors. In our study this could potentially have biased the sample due to attrition. If we had collected these data, the results could to some degree have been statistically corrected for these factors.

### Clinical implications

The main clinical implications of the present study are that IQ moderates outcome as measured with CGAS and that patients with the highest initial HoNOSCA scores show the greatest improvements. These results are potentially important as background information when interpreting changes in CGAS and HoNOSCA scores in ordinary clinical practice.

In spite of the findings that low IQ is a risk factor for mental health disorders [6-10], most intervention studies use IQ < 80 as an exclusion criterion [44-46]. In a psychodynamic psychotherapy study on child internalizing disorders, the cut off for exclusion was as high as IQ < 90 [47]. Since children and adolescents with low IQ systematically have been excluded form most outcome studies, there is a limited knowledge of whether they benefit from treatment in outpatient clinics or not. To make sure that children with low IQ receive effective help for their mental health problems, it is particularly important to apply systematic outcome evaluations on this group of children and adolescents to evaluate the effect of treatment.

## Conclusion

The results from the present study showed that both symptom load and general functioning among children and adolescents receiving treatment in the outpatient clinics improved. FSIQ and PIQ was a moderator of change in general functioning and symptom load, respectively. This implies that patients with higher IQ scores had a steeper improvement slope than those with lower scores. The patients with the highest initial symptom loads showed the greatest improvement, this pattern was not found in the improvement of general functioning.

## Abbreviations

HoNOSCA: The Health of the Nation Outcome Scales for Children and Adolescents; CGAS: The Children’s Global Assessment Scale; WISC-III: The Wechsler Intelligence Scale for Children—Third Edition; FSIQ: Full Scale IQ; VIQ: Verbal IQ; PIQ: Performance IQ; SDQ: The Strength and Difficulties Questionnaire.

## Competing interests

The authors declare that they have no competing interests.

## Authors’ contributions

BM was responsible for data analysis and manuscript writing. PHB participated in the data analysis and commented on the written drafts. MM and KW supervised the writing and commented on the written drafts. ME, KHB and SK designed and planned the study as well as they participated in the collection of data and commented on the written drafts. SK was the responsible manager of the study. All authors read and approved the final manuscript.
